# The regulatory role and mechanism of TRPV3 on apoptosis and inflammation in osteoarthritis

**DOI:** 10.17179/excli2024-8109

**Published:** 2025-03-03

**Authors:** Sahar Ghafari, Amin Moqadami, Mohammad Khalaj-Kondori

**Affiliations:** 1 Department of Animal Biology, Faculty of Natural Sciences, University of Tabriz, Tabriz, Iran

**Keywords:** osteoarthritis, interleukin-1 beta, apoptosis, inflammation, TRPV3

## Abstract

Osteoarthritis (OA) is one of the most common forms of degenerative joint disease characterized by persistent pain, inflammation of the joints, and restricted range of motion among the elderly worldwide. Interleukin-1 beta (IL-1β) is increased in the injured joints and contributes to the OA pathobiology by inducing chondrocyte apoptosis and inflammation. Transient receptor potential (TRP) ion channels have recently been reported as potential players in the modulation of apoptosis and inflammation. Here, we aimed to understand the regulatory role and effect of TRPV3 on apoptosis and inflammation in osteoarthritis by using C28/I2 chondrocyte cells as a model. Chondrocytes were transfected with TRPV3-specific siRNA for 24 hours and then stimulated with IL-1β in vitro. Cell cycle progression and apoptosis were evaluated with flow cytometry. The levels of TRPV3, apoptotic (Bax, Caspase-3, and Bcl-2), and inflammatory (iNOS, COX-2) genes were measured by quantitative real-time polymerase chain reaction (qRT-PCR) and confirmed with western blot. Treatment of the C28/I2 chondrocyte cells with IL-1β resulted in the over-expression of TRPV3, induction of apoptosis, and over-expression of inflammation indices. Knockdown of TRPV3 significantly reduced the expression of Bax and Caspase 3 proapoptotic factors while increasing the expression of the Bcl-2 antiapoptotic factor in the mRNA and protein levels in the IL-1β-stimulated cells. Its knockdown also decreased the expression of the inflammatory factors iNOS and COX-2 in mRNA and protein levels, confirming that TRPV3 knockdown hinders apoptosis and inflammation in IL-1β-stimulated chondrocytes. In conclusion, we demonstrated that si-TRPV3 treatment significantly mitigates IL-1β-related effects on the C28/I2 chondrocyte cells. These findings suggested that TRPV3 could be an effective target for the treatment of OA.

See also the graphical abstract(Fig. 1).

## Introduction

Osteoarthritis (OA) is a prevalent degenerative joint disease that affects the aged globally. It is typified by joint inflammation, ongoing discomfort, and limited range of motion (Moqadami et al., 2024[24], Gupta et al., 2005[13]). As OA progresses, it becomes a substantial financial burden due to joint dysfunction and pain (Fusco et al., 2017[11]). Current research indicates that this disease is characterized by the simultaneous occurrence of joint dysfunction, chondrodegeneration, and low-grade chronic inflammation (Robinson et al., 2016[28]; Abramoff and Caldera, 2020[1]; Hunter and Bierma-Zeinstra, 2019[16]). There are various risk factors that cause OA, including aging, obesity, and trauma (Wojdasiewicz et al., 2014[35]). The only cells in cartilage tissue that are significant regulators of cartilage degradation are chondrocytes, which are involved in physiological processes like apoptosis, inflammation, and autophagy (Katz et al., 2021[18]). Recent research has shown that inflammatory reactions and chondrocyte apoptosis play significant roles in the etiology of OA (Berenbaum, 2013[3]; Hwang and Kim, 2015[17]).

Interleukin-1β (IL-1β) intimately associates with the formation and progression of OA, at least partly by generating inflammatory responses. Its level goes up in OA and causes OA progression (Xu et al., 2021[38]; Wu et al., 2020[36]). In addition, chondrocytes treated with IL-1β may trigger the release of cyclooxygenase-2 (COX-2) and inducible nitric oxide synthase (iNOS), which would result in the synthesis of prostaglandin E2 (PGE2) and nitric oxide (NO) (Chen et al., 2019[7]). As a result, IL-1β is frequently utilized for creating OA cell models *in vitro*. 

The progression of OA is intimately linked to chondrocyte apoptosis. As is commonly known, elevated IL-1β causes chondrocytes to undergo apoptosis by upregulating pro-apoptotic and downregulating anti-apoptotic proteins, which speeds up the deterioration of cartilage (Wang et al., 2014[33]; Chen and Zhang, 2024[6]). Pro-apoptotic factors Bax, cleaved caspase-3, and cleaved caspase-7 are expressed more than usual in OA chondrocytes, but Bcl-2, an anti-apoptotic factor, is expressed less than usual (Na et al., 2012[27]; Musumeci et al., 2014[26]).

One important intracellular second messenger considered a crucial regulator of cell viability is calcium ion (Ca^2+^) (Bouron et al., 2016[4]). It's well knowledge that prolonged intracellular Ca^2+^ elevation can cause reactive oxygen species (ROS) generation that is dependent on Ca^2+^ entry, mitochondrial depolarization, and, ultimately apoptosis (Kovac et al., 2014[20]; Llorente-Folch et al., 2015[22]; Kim et al., 2011[19]). Transient receptor potential (TRP) ion channels represent a significant family of membrane-associated cation channels characterized by their high permeability to Ca^2+^ (Hasan and Zhang, 2018[15]). Consequently, the activation of TRP channels functions as a crucial Ca^2+^ entry pathway, influencing intracellular Ca^2+^ fluctuations and subsequent signaling pathways (Mulier et al., 2017[25]). Recent evidence has indicated the significant role of excessive Ca^2+^ influx via TRP ion channels in cellular mortality (Fliniaux et al., 2018[10]). Transient receptor potential vanilloid (TRPV), a type of TRP channel, has been identified as an essential participant in the pathophysiology of OA in recent years. These channels have a role in mechanotransduction, inflammation, and pain perception, among other features of osteoarthritis (Chen et al., 2024[5]). A Ca^2+^-permeable nonselective cation channel known as transient receptor potential vanilloid-3 (TRPV3) is sensitive to heat stimulation in the 31-39 °C range. It is essential for the feeling of warmth, the transmission of pain, the physiology of the skin, inflammation, and other illnesses (Su et al., 2023[31]). This ion channel mainly mediates pain and inflammation (Su et al., 2023[31]) and is widely expressed in neuronal and non-neuronal cells (Luo and Hu, 2014[23]). 

Nevertheless, elucidating the connection between TRPV3 and chondrocyte apoptosis and inflammation has not received much focus until now. We thus set out to determine whether or not TRPV3 would have a regulatory role in modulating IL-1β-induced inflammation and death in C28/I2 chondrocyte cells.

## Materials and Methods

### Cell culturing and treatment 

The human chondrocyte cell line C28/I2 purchased from the National Cell Bank of Pasteur Institute, Iran, Tehran, was cultivated in Dulbecco's modified Eagle's medium (Hyclone, Logan, UT, USA) containing 10 % fetal bovine serum (FBS) (Anacell, Tehran, Iran) and 1 % penicillin and streptomycin (Bioideaco, Tehran, Iran). The culture was maintained at 37 °C in a humidified environment with 5 % CO_2_. Every two days, a new medium was used. Four groups were investigated: A: Control (cells with no treatment), B: IL-1β (cells stimulated with IL-1β), C: IL-1β+Si-TRPV3 (cells stimulated with IL-1β and transfected with TRPV3-specific siRNA), and D: IL-1β+Si-NC (cells stimulated with IL-1β and transfected with siRNA negative control).

### Cell transfection

Metabion, Germany, generated the small interfering RNA (siRNA) and controls against TRPV3. The sequences were as follows: si-TRPV3 sense, 5´-GGGCGAACAUGCUCUACUAUATT-3´, and antisense, 5´-UAUAGUAGAGCAUGUUCGCCCTT-3´, and Si-NC sense, 5´-AACAGCUUCGGGUACAAGUCUTT-3´, and Si-NC antisense, 5´-AGACUUGUACCCGAAGCUGUUTT-3´. For cell transfection, 2.5×10^5 ^C28/I2 cells per well were seeded into 6-well plates and allowed to grow to a cell density of 70 %. Next, 20 nM oligonucleotides were transfected using lipofectamine®2000 (Invitrogen; Thermo Fisher Scientific, USA). After six hours, the medium was changed to a new one, and the culture was allowed to continue for a full twenty-four hours. Following transfection, cells were treated for 24 hours with 10 ng/ml IL-1β ((Purity: >95 %) (Sino Biological, Beijing, China)) (Liu et al., 2023[21]) before being utilized in the following tests.

### Cell viability assay 

Human C28/I2 chondrocytes in the logarithmic phase were taken, seeded into 96-well plates (1 × 10^4^ cells per well), and placed in an incubator. Subsequently, three repetitions of each set of cells treated with various substances were added (a blank control group received an equivalent amount of PBS). Following a 24-hour culture period, 50 μL MTT (Sigma, St. Louis, MO, USA) (5 g/L) was added, and the cells were then cultivated for an additional 4 hours at 37 °C. Afterward, we removed the supernatant, filled each well with 150 μL of DMSO (Sigma, St. Louis, MO, USA), and gave it a good shake using a plate shaker. Following the crystals' dissolution, the OD value of each well was measured at 490 nm using a microplate reader.

### RNA extraction, cDNA synthesis, and quantitative real-time PCR (qRT-PCR)

The cells were separated from the plates using 0.25 % EDTA-trypsin (Sigma, St. Louis, MO, USA). As directed by the manufacturer, total RNA was extracted using column RNA extraction (DENAzistAsia, Mashhad, Iran). The RNA samples were quantified and qualified with a NanoDrop spectrophotometer and 2 % agarose gel electrophoresis. The cDNA was synthesized using the Parstous kit following the kit instructions and used in qRT-PCR. The housekeeping gene U6 was used to normalize the expression data. The qRT-PCR protocol involved 15 min at 95 °C for primary denaturation, which was contained with 40 cycles at 95 °C for 15 sec, annealing temperature for 30 sec, and at 72 °C for 30 sec, and finished with a final extension at 72 °C for 5 min. The 2-∆∆Ct method was used to determine the fold change of each mRNA expression. Table 1(Tab. 1) listed the primer sequences and their corresponding amplification products.

### Apoptosis evaluation 

To study the impacts of TRPV3 knockdown on apoptosis, we utilized the Annexin V-FITC/PI apoptosis kit (ImmunoStep, Salamanca, Spain). After cultivating 2.5×10^5^ C28/I2 cells in 6-well plates, all four groups underwent a 24-hour treatment, were re-suspended in 197 μL binding buffer, and were stained with 3 μL Annexin V-FITC and 1 µL PI in a dark, room-temperature environment for 15 minutes. The cells were then examined by flow cytometry (Becton Dickinson, San Jose, CA, USA). Using the FlowJo analytic software version 10, the percentages of early and late apoptotic cells were calculated.

### Cell cycle analysis 

After plating 2.5×10^5^ C28/I2 cells per well per 2 mL on a six-well culture plate, the cells were incubated for 24 hours at 37 °C in a CO_2_ incubator. Trypsin was used in this instance to separate the cells after 24 hours, and they were then washed with cold phosphate-buffered saline (PBS) and preserved in 90 % ethanol at -20 °C for the night. After centrifuging the ethanol-fixed cells and washing them with PBS, they were stained with a PI and RNase A (Simbiolab, Mashhad, Iran) solution at final concentrations of 50 mg/mL and 20 mg/mL, respectively. The BD FACSCalibur cytometer (Becton Dickinson, San Jose, CA, USA) was used for assessing the cell cycle phases.

### Western blot

The cells were lysed using a lysis buffer composed of 0.1 % SDS, 1 % Triton X-100, 1.5 M NaCl, 1 mM EDTA, and 20 mM Tris HCl (pH 7.4), supplemented with 1 % phenylmethanesulfonyl fluoride. Total protein was purified by centrifugation at 16,000 g for 10 minutes at 4 °C. The protein concentration was determined using the DC protein assay from BioRad. A constant voltage of 80 mV was applied for 30 minutes, followed by 120 mV for 120 minutes, to separate 30 µg of protein from each group via electrophoresis. The proteins were then transferred to PVDF membranes using the Bio-Rad TransBlot system at 100 mV for 120 minutes (Bio Rad Laboratories, Inc.). A solution of 5 % non-fat dried milk in 10 mM Tris HCl, 0.2 % Tween-20 (TBST), and 0.15 M NaCl was utilized to block the membrane for 2 hours at room temperature. The membrane underwent incubation with primary antibodies targeting TRPV3 (mouse; cat. no. sc-45407), Bax (mouse; cat. no. sc-7480), Bcl-2 (rabbit; cat. no. sc-492), caspase-3 (mouse; cat. no. sc7272), iNOS (mouse; cat. no. sc-7271), COX-2 (mouse; cat. no. sc-514489), and β-Actin (mouse; cat. no. sc-47778) overnight at 4 °C. The membrane underwent incubation with mIgG κBP-HRP (mouse; cat. no. sc-516102) and mouse anti-rabbit IgG HRP (mouse; cat. no. sc-2357) secondary antibodies at room temperature for 2 hours, following three rinses with TBST for 5 minutes each. The membrane underwent treatment with an ECL chemiluminescence kit and was subsequently analyzed using the Mini-PROTEAN Tetra Vertical Electrophoresis Cell (Bio-Rad). Densitometry quantification of the images was performed using ImageJ v1.8.0 software from the National Institutes of Health.

### Statistical analysis 

Data analysis was conducted using GraphPad Prism (San Diego, CA, USA) version 9.0 and SPSS version 26. Data normalization was performed as necessary. A two-tailed t-test was conducted to compare the two groups, with the data presented as the mean ± SD. FlowJo v10.8 was employed to evaluate the data obtained from flow cytometry assays. Image processing and quantification of the western blotting data were done using ImageJ v1.8.0 software. Statistical significance was defined as P < 0.05.

## Results

### TRPV3 expression and its knockdown in the IL-1β-treated C28/I2 chondrocyte cells 

**Figure 2:**
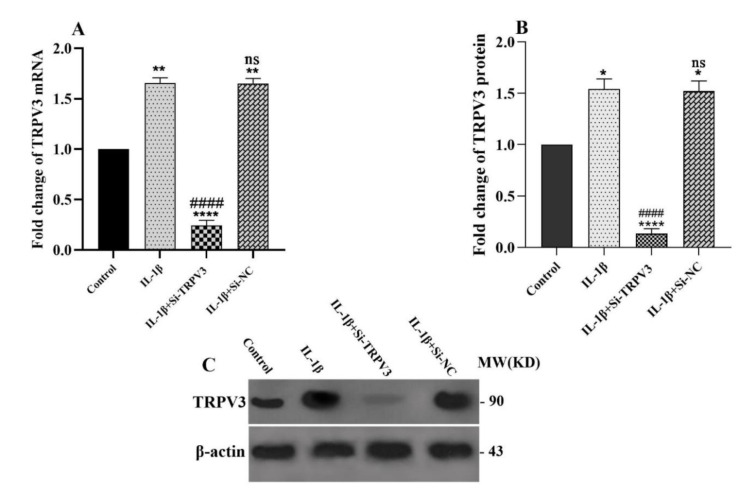
Expression of TRPV3 in IL-1β treated C28/I2 chondrocyte cells. (A) expression of TRPV3 in mRNA level was assessed with qRT-PCR in the C28/I2 cells treated with IL-1β, Si-TRPV3+IL-1β, or Si-NC +IL-1β (siRNA negative control+IL-1β) as siRNA negative control, or no treatment as control. (B) expression of TRPV3 in protein level was evaluated by western blot. (C) Selected blots reflect corresponding protein levels. Data are shown as mean ± SD. All experiments were evaluated three times. Significance: ns > 0.05, *p < 0.05, **p < 0.01, and ***p < 0.001 vs. control and ^#^p < 0.05, ^##^p < 0.01, ^###^p < 0.001 and, ^####^p < 0.0001 vs. IL-1β

To investigate the role of TRPV3 in OA, we assessed its expression in the IL-1β-treated C28/I2 chondrocyte cells by qRT-PCR and western blot. As shown in Figure 2(Fig. 2), IL-1β-treated C28/I2 chondrocyte cells upregulated the expression of TRPV3 in both mRNA and protein levels, implying that IL-1β enhances TRPV3 expression in chondrocyte cells. We applied TRPV3-specific siRNA (si-TRPV3) to knockdown its expression. As Figure 2(Fig. 2) shows, transfection of the IL-1β-treated C28/I2 cells with si-TRPV3 effectively repressed TRPV3 expression in the mRNA and protein levels. 

### Knockdown of TRPV3 accelerated the proliferation and inhibited the IL-1β- induced apoptosis of the C28/I2 cells

**Figure 3:**
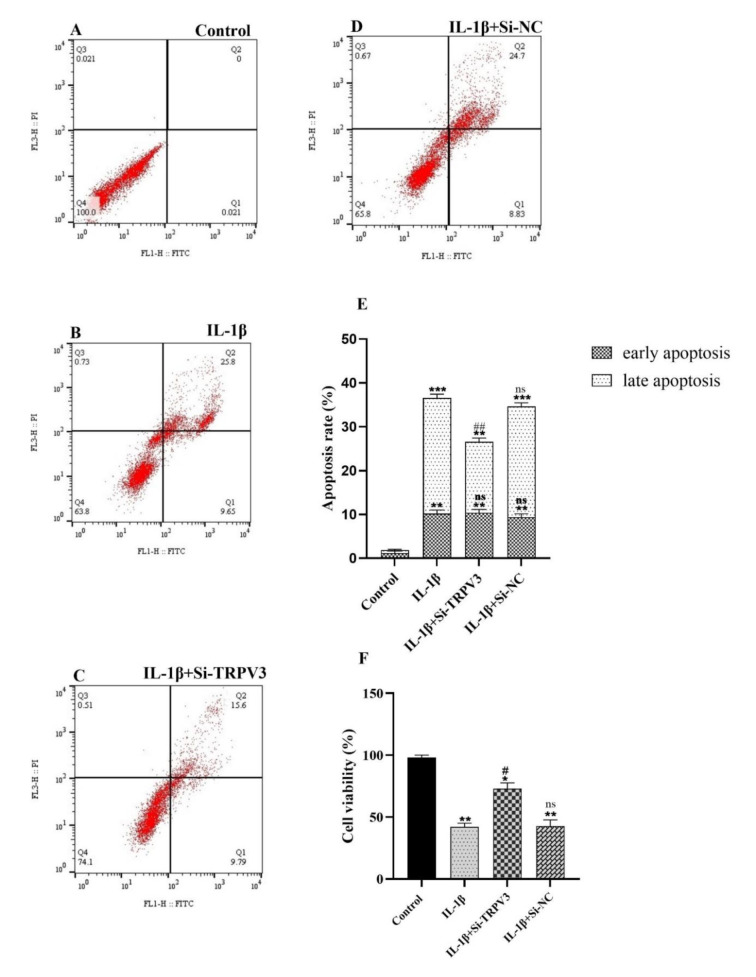
Examination of cellular apoptosis. The impact of Si-TRPV3 on apoptosis of IL-1β-treated C28/I2 chondrocyte cells was assessed using flow cytometry. (A) Control group; (B) C28/I2 cells were treated with 10 ng/mL of IL-1β; (C) cells received Si-TRPV3 treatment for 6 hours, followed by the addition of IL-1β (10 ng/mL) for 24 hours; (D) cells were treated with Si-NC in conjunction with IL-1β, (E) A graph illustrating the average percentage of early and late apoptosis for each treatment in the apoptosis assay. (F) Cell viability was evaluated with the MTT test. The error bars represent the standard deviation (SD) of three independent investigations. Significance: ns > 0.05, *p < 0.05, **p < 0.01 and ****p < 0.0001 vs. Control group and ^#^p < 0.05, ^##^p < 0.01 and ^####^p < 0.0001 vs. IL-1β group

Chondrocyte cell viability was assessed after treatments of the cells with IL-1β, Si-TRPV3+IL-1β, and siRNA negative control +IL-1β (Si-NC+IL-1β). The cell viability was significantly decreased in response to the IL-1β treatment compared to the control (Figure 3F(Fig. 3)). To verify whether the cytotoxicity induced by IL-1β occurred via apoptosis, we employed flow cytometry to measure the apoptosis of the C28/I2 cells treated with IL-1β. Figure 3A and B(Fig. 3) demonstrate that IL-1β significantly induced apoptosis in C28/I2 cells, with 9.65 % early and 25.8 % late apoptosis compared to the control group. To understand whether TRPV3 knockdown could affect chondrocyte cell viability, C28/I2 cells were pretreated with Si-TRPV3 or Si-NC for 6 hours before treatment with IL-1β. As Figure 3F(Fig. 3) shows the cell viability was significantly increased in response to the knockdown of TRPV3. Besides, the findings indicated a notable decrease in the percentage of apoptotic chondrocytes in the Si-TRPV3+IL-1β treated cells (early apoptosis 9.79 % and late apoptosis 15.6 %) in comparison to the IL-1β-treated cells (early apoptosis 9.65 % and late apoptosis 25.8 %) (Figure 3C(Fig. 3)). However, treatment of the cells with Si-NC +IL-1β resulted in non-significant differences compared to the IL-1β-treated cells (Figure 3D(Fig. 3)). Figure 3E(Fig. 3) presents the results derived from three independent experimental replicates.

### Impact of TRPV3 knockdown on the C28/I2 cell cycle progression 

**Figure 4:**
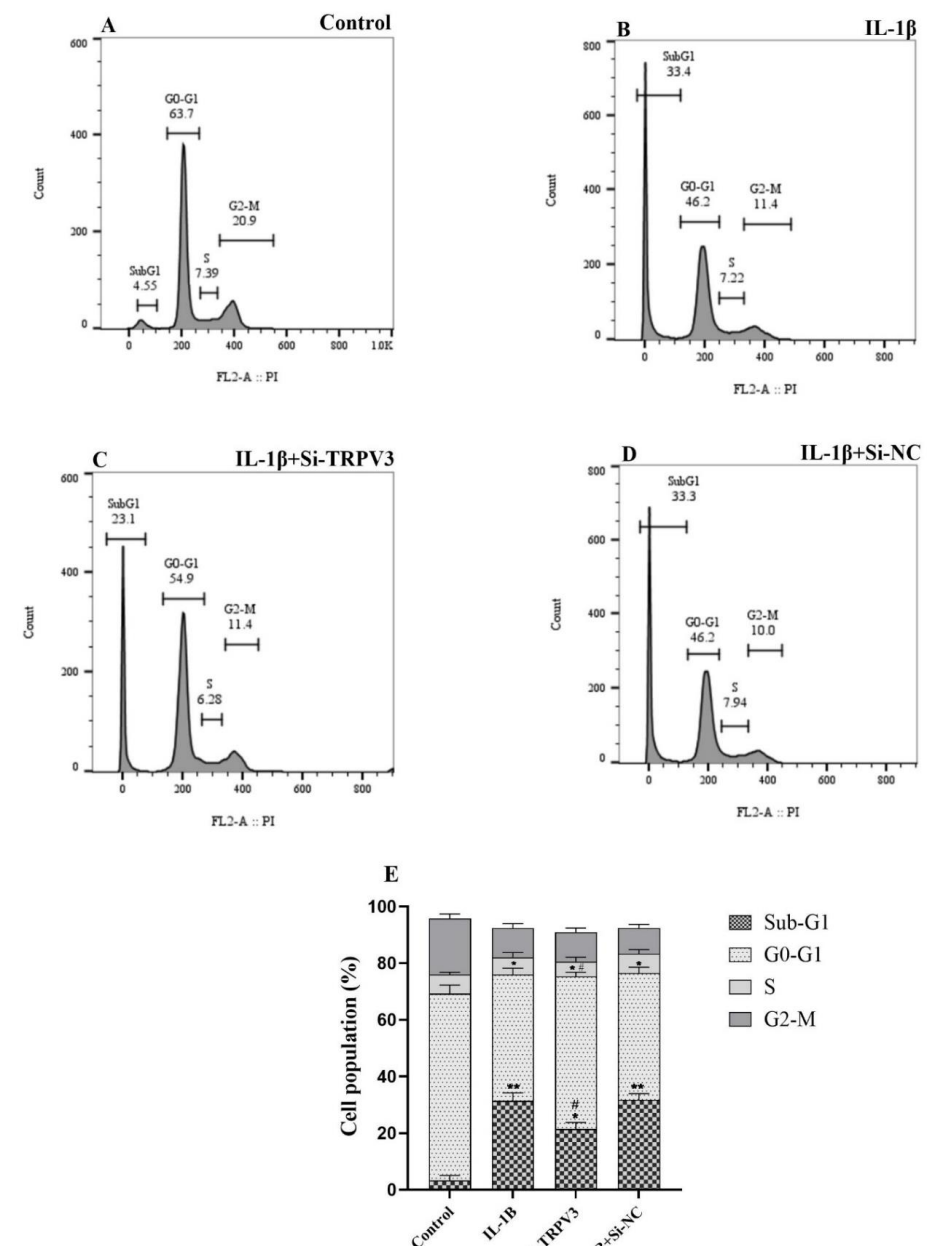
Cell cycle phase distribution analysis in control and treated C28/I2 chondrocyte cells. (A) Distribution of cell cycle phases in control cells. (B) Following treatment with a 10 ng/ml concentration of IL-1β, a significant increase in the number of cells in the sub-G1 phase is observed, indicating the presence of apoptotic cells. (C) After 24-hour treatment with 10 ng/ml Si-TRPV3+IL-1β, the number of apoptotic cells was significantly decreased compared with the IL-1β-only group. (D) After 24-hour treatment with 10 ng/ml Si-NC+IL-1β, the count of apoptotic cells was not significantly different from that of the IL-1β-only group. (E) Shows findings from three independent tests for the distribution of cell cycle phases. Error bars show the SD (standard deviation) of three separate experiments. Significance: *p < 0.05, **p < 0.01, ***p < 0.001, ****p < 0.0001 vs. control and ^#^p < 0.05, ^##^p < 0.01, ^###^p < 0.001 and ^####^p < 0.0001 vs. IL-1β.

Flow cytometry assessed the effect of Si-TRPV3+IL-1β, IL-1β, and Si-NC +IL-1β interventions on the cell cycle progression of C28/I2 chondrocyte cells. The results indicated that treatment with IL-1β considerably elevated the sub-G_1_ cell phase population to 33.4 % compared to the untreated control cells, which exhibited 4.55 %. Conversely, the sub-G_1_ cell phase population of the Si-NC +IL-1β group exhibited an insignificant change (33.3 %) in comparison to the IL-1β treatment group (33.4 %) (Figure 4B and D(Fig. 4)). The Si-TRPV3+IL-1β treatment group exhibited a reduction in the sub-G_1_ cell phase population (23.1 %) in comparison to the IL-1β treated cells (33.4 %) (Figure 4B and C(Fig. 4)). Figure 4E(Fig. 4) delineates the findings derived from three independent experiments. 

### Effect of TRPV3 knockdown on the expression of apoptotic genes

**Figure 5:**
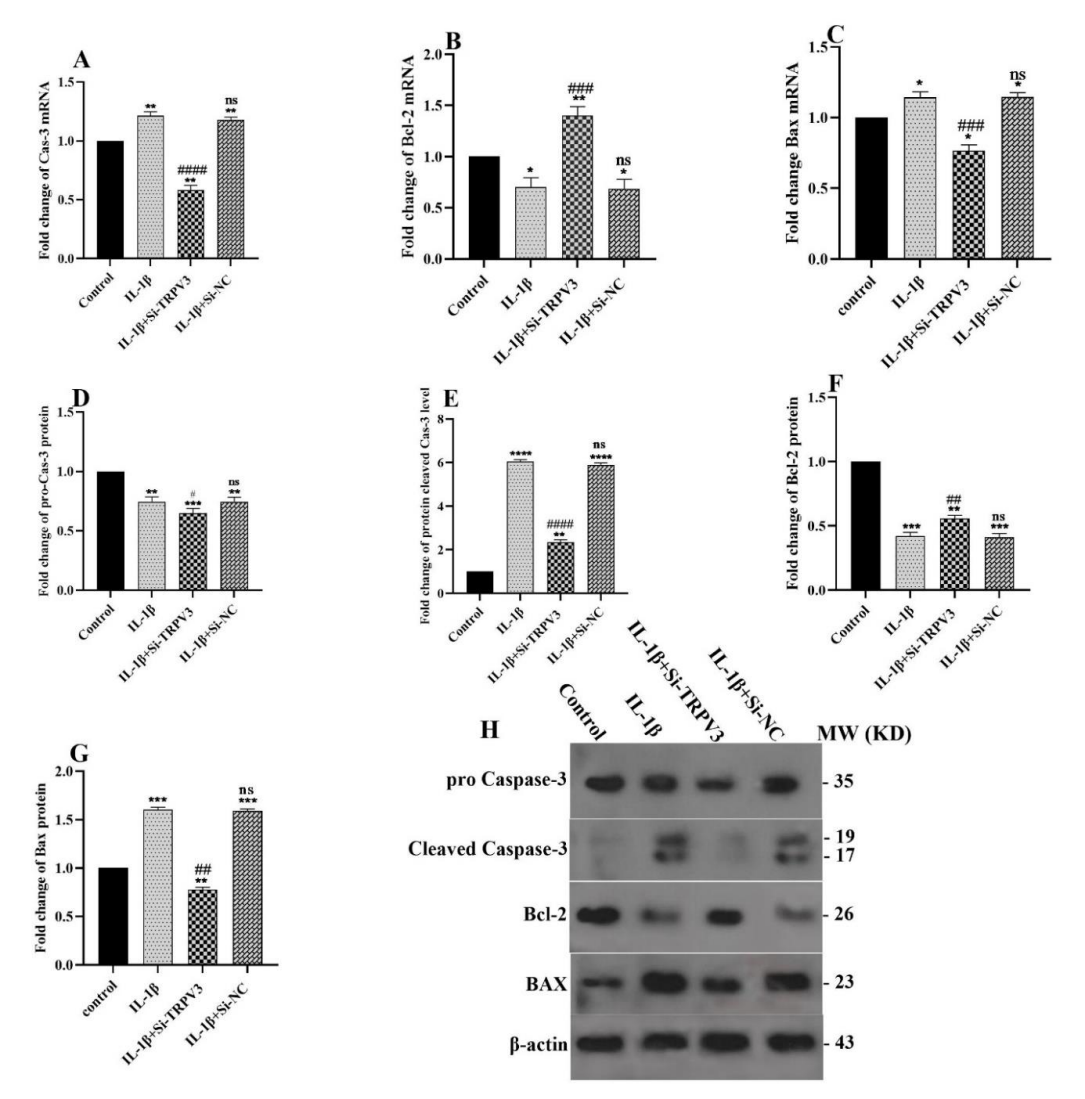
The effect of Si-TRPV3 on the mRNA and protein expression of apoptosis genes. The mRNA expression levels of (A) Caspase-3, (B) Bcl-2, and (C) Bax were evaluated using qRT-PCR. Protein levels of (D) pro-Caspase-3, (E) Cleaved Caspase-3, (F) Bcl-2, and (G) Bax were assessed using western blot analysis. (H) The selected blots indicate the levels of the corresponding proteins. Data are shown as mean ± SD. All experiments were examined three times. Significance: ns > 0.05, *p < 0.05, **p < 0.01, and ***p < 0.001 vs. control and ns > 0.05, ^#^p < 0.05, ^##^p < 0.01 and, ^###^p < 0.001 vs. IL-1β

Quantitative Real-time PCR and western blotting were used to assess the expression of Bax, Caspase-3, and Bcl-2. Figure 5(Fig. 5) illustrates how IL-1β treatment significantly increased the mRNA and protein levels of pro-apoptotic Bax and Caspase-3 genes and significantly decreased the mRNA and protein levels of anti-apoptotic Bcl-2. Si-TRPV3 treatment notably elevated Bcl-2 expression (anti-apoptotic) and reduced the expression of Bax and Caspase-3 (pro-apoptotic) genes. Western blotting validated these effects at the protein level (Figure 5A-H(Fig. 5)).

### Effect of TRPV3 knockdown on the expression of inflammation genes

**Figure 6:**
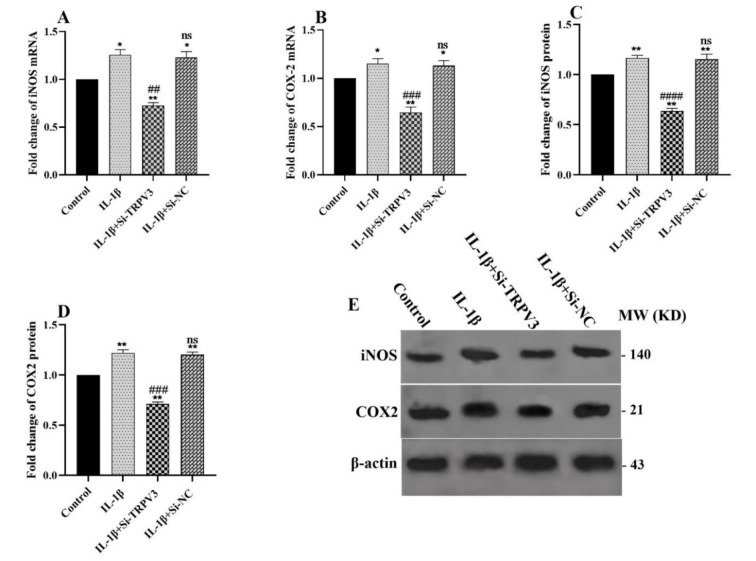
Effects of TRPV3 knockdown on the expression of inflammation genes. The cells underwent pretreatment with Si-TRPV3 for 6 hours before a subsequent treatment with 10 ng/ml IL-1β for 24 hours. The mRNA expression levels of (A) iNOS and (B) COX-2 were assessed by qRT-PCR, and the protein expression levels of (C) iNOS and (D) COX-2 were measured by western blot, respectively. (E) Selected blots reflect corresponding protein levels. Data are shown as mean ± SD. All experiments were examined three times. Significance: ns > 0.05, *p < 0.05, **p < 0.01, ***p < 0.001, ****p < 0.0001 vs. control and ns > 0.05, ^#^p < 0.05, ^##^p < 0.01, ^###^p < 0.001 and ^####^p < 0.0001 vs. IL-1β

Measurements of iNOS and COX-2, mRNA and protein levels, were also conducted using qRT-PCR and western blotting, respectively, to ascertain the impact of TRPV3 knockdown on osteoarthritis inflammation. Our findings indicated that IL-1β treatment elevated both mRNA and protein levels of iNOS and COX-2 expression; however, the treatment of chondrocyte cells with Si-TRPV3 considerably inhibited the expression of these genes (Figure 6A-E(Fig. 6)).

See also the supplementary data.

## Discussion

Osteoarthritis (OA) is a prevalent degenerative joint disease marked by the progressive degeneration of articular cartilage, primarily driven by increased chondrocyte apoptosis (Vina and Kwoh, 2018[32]). In recent years, transient receptor potential vanilloid (TRPV) channels have emerged as key players in OA pathogenesis (Chen et al., 2024[5]). Halonen et al. demonstrated that IL-1β increased the expression of TRPV3 in chondrocytes, which aligns with our findings (Halonen et al., 2023[14]). IL-1β is a critical inflammatory factor in the early stages of osteoarthritis, produced in damaged and degenerated joints, and it can be utilized to establish the OA cell model *in vitro* (Zhang et al., 2019[40]). Consequently, investigating specific processes related to IL-1β induced chondrocyte apoptosis and inflammation is crucial for advancing targeted treatments for osteoarthritis. In this study, we demonstrate that IL-1β elevates TRPV3 levels and activates apoptosis and inflammation. Knockdown of TRPV3 level or inhibiting TRPV3-related activity demonstrated a protective effect against chondrocyte apoptosis and inflammation.

Chondrocyte apoptosis and the inflammatory response are critical pathogenic processes in the advancement of osteoarthritis, and effective management in this process is crucial for the treatment of OA. Apoptosis is vital for appropriate development, chemically induced cell death, and regular cell turnover (Xiao et al., 2023[37]). Abnormal apoptosis consequently contributes to various human disorders, including neurodegenerative diseases, musculoskeletal degeneration, and numerous cancer types (Elmore, 2007[9]; Singh et al., 2019[30]). We hypothesized that the knockdown of TRPV3 may mitigate the effects induced by IL-1β in patients with osteoarthritis. We examined this hypothesis by conducting an *in vitro *study using an OA cell model, treating C28/I2 chondrocyte cells with IL-1β. Figure 3(Fig. 3) illustrates that IL-1β induced apoptosis and inflammation in C28/I2 chondrocyte cells. Furthermore, treatment of C28/I2 cells with si-TRPV3 significantly reduced the number of IL-1β-induced apoptotic cells. This observation suggests that the downregulation of TRPV3 may effectively protect chondrocyte cells from IL-1β-induced apoptosis. The cell cycle phase analysis verified these observations. Figure 4(Fig. 4) demonstrates that si-TRPV3 effectively decreased the proportion of the Sub-G_1_ cell population, which was markedly elevated in the IL-1β treated cells. 

The analysis of pro-apoptotic Bax, Caspase-3, and anti-apoptotic Bcl-2 factors further substantiated the anti-IL-1β effects of Si-TRPV3. Figure 5(Fig. 5) demonstrates that the knockdown of TRPV3 markedly reduced the IL-1β-induced production of Bax and caspase-3 while enhancing Bcl-2 expression levels at both the mRNA and protein levels. A recent study has shown that TRPV channels are crucial in the development of osteoarthritis (OA) (Chen et al., 2024[5]). Our findings align with the prior report by Wang et al., which demonstrated that TRPV3 inhibition decreased cardiomyocyte apoptosis (Wang et al., 2021[34]). Furthermore, Yin et al. demonstrated that the TRPA1 inhibitor could diminish the abnormal expressions of mitochondrial pro-apoptotic proteins, such as Bax, cytochrome c, PARP, caspase-3, and caspase-9, in an IL-1β-induced apoptosis model (Yin et al., 2018[39]).

We assessed the impact of TRPV3 downregulation on inflammatory mediators, including iNOS and COX-2, in IL-1β-stimulated chondrocytes, as their dysregulation contributes to the pathobiology of osteoarthritis. Chow et al. demonstrated that the nitric oxide route (inducible nitric oxide synthase (iNOS), nitric oxide (NO)) and the prostacyclin pathway (cyclooxygenase-2 (COX-2), prostaglandin E2 (PGE2)) are essential to the pathophysiology of osteoarthritis (Chow and Chin, 2020[8]). IL-1β enhances the expression of both iNOS and COX-2 in osteoarthritis, resulting in elevated production of NO (Sasaki et al., 1998[29]) and PGE2 (Goggs et al., 2003[12]), respectively. According to other research (Wu et al., 2020[36]; Ansari and Haqqi, 2016[2]), treating C28/I2 chondrocyte cells with IL-1β resulted in a substantial increase in the expression of iNOS and COX-2. These findings corroborate prior assertions that Si-TRPV3 can potentially suppress various inflammation-related genes (Wang et al., 2021[34]). 

In conclusion, this study has verified that IL-1β induces chondrocyte apoptosis and the upregulation of inflammation mediators (iNOS, COX-2), which contribute to cartilage deterioration. We demonstrated that treatment with Si-TRPV3 substantially reduces the expressions of iNOS, COX-2, and apoptotic factors and ameliorates the effects of IL-1β on the C28/I2 chondrocyte cells. These results indicated that TRPV3 may be a viable target for the treatment of OA.

## Declaration

### Acknowledgment

The authors appreciate the research deputy of the University of Tabriz for general support of the study.

### Ethics approval and consent to participate

Not applicable.

### Availability of data and material

Data are available upon reasonable request.

### Competing interests

The authors declare no competing interest.

### Funding

No funds, grants, or other support were received.

### Authors contributions

SG, AM, and MKK, designed the study. SG collected data and wrote the manuscript. MKK supervised, directed, and managed the study. SG, AM, and MKK approved the version to be published.

## Supplementary Material

Supplementary data

## Figures and Tables

**Table 1:**
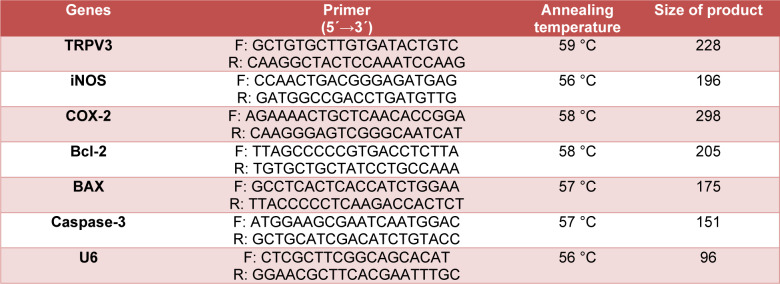
The sequences of primers and their amplification product sizes

**Figure 1:**
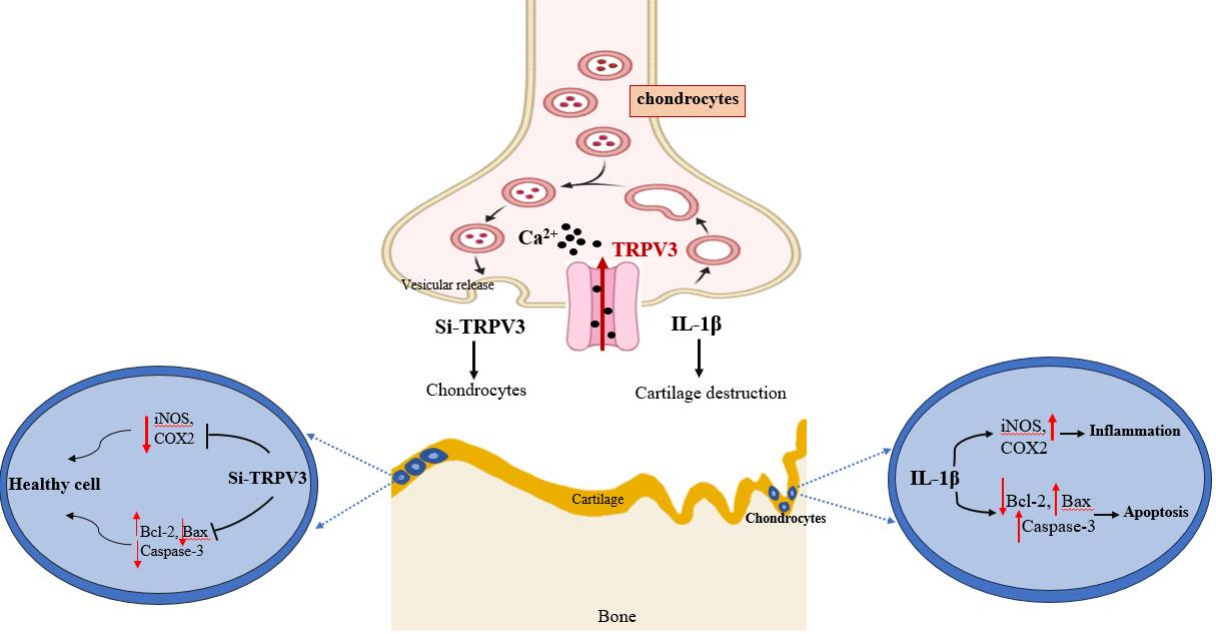
Graphical abstract. Osteoarthritis is a degenerative joint condition associated with aging, and one of the primary cytokines that cause it is interleukin-1beta, which induces apoptosis and inflammation. In this study, we investigated the regulatory role and mechanism of a Ca^2+^-permeable nonselective cation channel known as transient receptor potential vanilloid-3 (TRPV3) on apoptosis and inflammation in osteoarthritis and transfected osteoarthritic cells with Si-TRPV3 reduced apoptosis and inflammation. These findings highlighted that TRPV3 could be an effective target for the treatment of OA.
