# Supplementary material for: The regulatory role and mechanism of TRPV3 on apoptosis and inflammation in osteoarthritis
Source: EXCLI J. 2025 Mar 3;24:325–38. doi: 10.17179/excli2024-8109 (PMC11956525; doi:10.17179/excli2024-8109)
Supplement: Supplementary data [file EXCLI-24-325-s-001.pdf]

EXCLI Journal 2025;24:325-338 – ISSN 1611-2156
Received: December 14, 2024, accepted: February 25, 2025, published: March 03, 2025

Supplementary data to:

Original article:

THE REGULATORY ROLE AND MECHANISM OF TRPV3 ON APOPTOSIS AND INFLAMMATION IN OSTEOARTHRITIS

Sahar Ghafari, Amin Moqadami, Mohammad Khalaj-Kondori*

Department of Animal Biology, Faculty of Natural Sciences, University of Tabriz, Tabriz, Iran

* **Corresponding author:** Mohammad Khalaj-Kondori, Department of Animal Biology, Faculty of Natural Sciences, University of Tabriz, Tabriz, Iran.
E-mail: khalaj@tabrizu.ac.ir

https://dx.doi.org/10.17179/excli2024-8109

This is an Open Access article distributed under the terms of the Creative Commons Attribution License (http://creativecommons.org/licenses/by/4.0/).

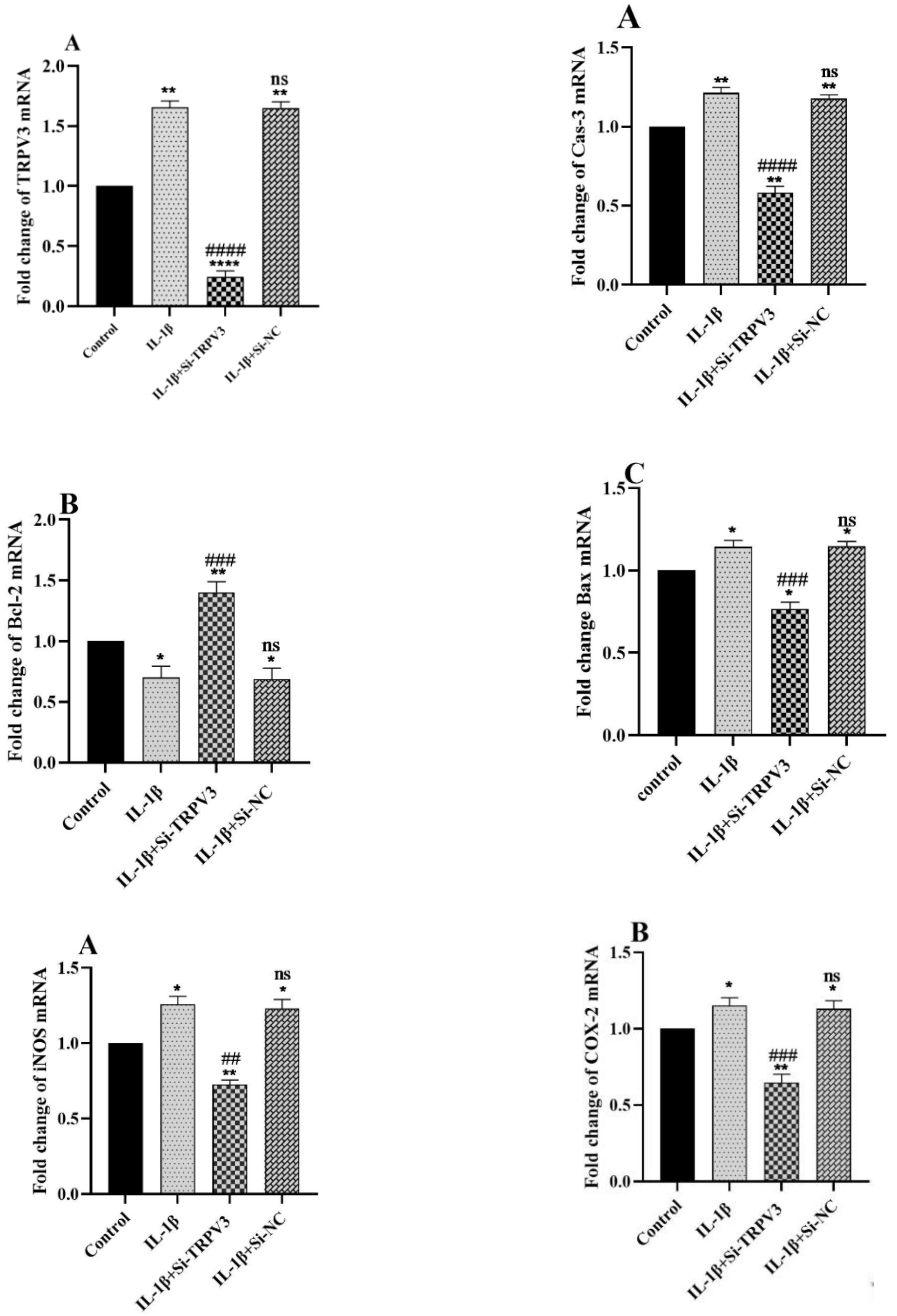

Figures 2, 5 and 6 (A, B, C, D, E, and F): Expression of TRPV3 in IL-1β treated C28/I2 chondrocyte cells and the effect of Si-TRPV3 on the mRNA expression of apoptosis and flammation genes

EXCLI Journal 2025;24:325-338 – ISSN 1611-2156
Received: December 14, 2024, accepted: February 25, 2025, published: March 03, 2025

Raw data to Figures 2, 5, and 6 (A, B, C, D, E, and F): The raw data indicating fold changes of studied genes of individual qRT-PCR include TRPV3, Caspase-3, Bax, Bcl-2, iNOS, and COX-2.

Genes	Groups	Experiments	Transcript Fold change ($2^{-\Delta\Delta CT}$) in treated cells
**TRPV3**	IL-1B	Experiment1	1.67018
		Experiment2	1.67189
		Experiment3	1.73261
	IL-1B+Si-TRPV3	Experiment1	0.23165
		Experiment2	0.20243
		Experiment3	0.30149
	IL-1B+Si-NC	Experiment1	1.65864
		Experiment2	1.60769
		Experiment3	1.70864
**Caspase-3**	IL-1B	Experiment1	1.21419
		Experiment2	1.18359
		Experiment3	1.25127
	IL-1B+Si-TRPV3	Experiment1	0.59049
		Experiment2	0.54138
		Experiment3	0.62257
	IL-1B+Si-NC	Experiment1	1.18099
		Experiment2	1.18188
		Experiment3	1.15265
**Bax**	IL-1B	Experiment1	1.148698
		Experiment2	1.136982
		Experiment3	1.182389
	IL-1B+Si-TRPV3	Experiment1	0.781284
		Experiment2	0.725327
		Experiment3	0.802563
	IL-1B+Si-NC	Experiment1	1.140764
		Experiment2	1.120529
		Experiment3	1.181671

EXCLI Journal 2025;24:325-338 – ISSN 1611-2156
Received: December 14, 2024, accepted: February 25, 2025, published: March 03, 2025

Genes	Groups	Experiments	Transcript Fold change ($2^{-\Delta\Delta CT}$) in treated cells
**Bcl-2**	IL-1B	Experiment1	0.712025
		Experiment2	0.613143
		Experiment3	0.792761
	IL-1B+Si-TRPV3	Experiment1	1.22264
		Experiment2	1.49136
		Experiment3	1.41283
	IL-1B+Si-NC	Experiment1	0.68302
		Experiment2	0.66903
		Experiment3	0.78329
**iNOS**	IL-1B	Experiment1	1.265757
		Experiment2	1.251834
		Experiment3	1.316873
	IL-1B+Si-TRPV3	Experiment1	0.768438
		Experiment2	0.721867
		Experiment3	0.729824
	IL-1B+Si-NC	Experiment1	1.239708
		Experiment2	1.173571
		Experiment3	1.294834
**COX-2**	IL-1B	Experiment1	1.164734
		Experiment2	1.108616
		Experiment3	1.204698
	IL-1B+Si-TRPV3	Experiment1	0.650671
		Experiment2	0.597219
		Experiment3	0.701284
	IL-1B+Si-NC	Experiment1	1.117287
		Experiment2	1.091429
		Experiment3	1.193421

EXCLI Journal 2025;24:325-338 – ISSN 1611-2156
Received: December 14, 2024, accepted: February 25, 2025, published: March 03, 2025

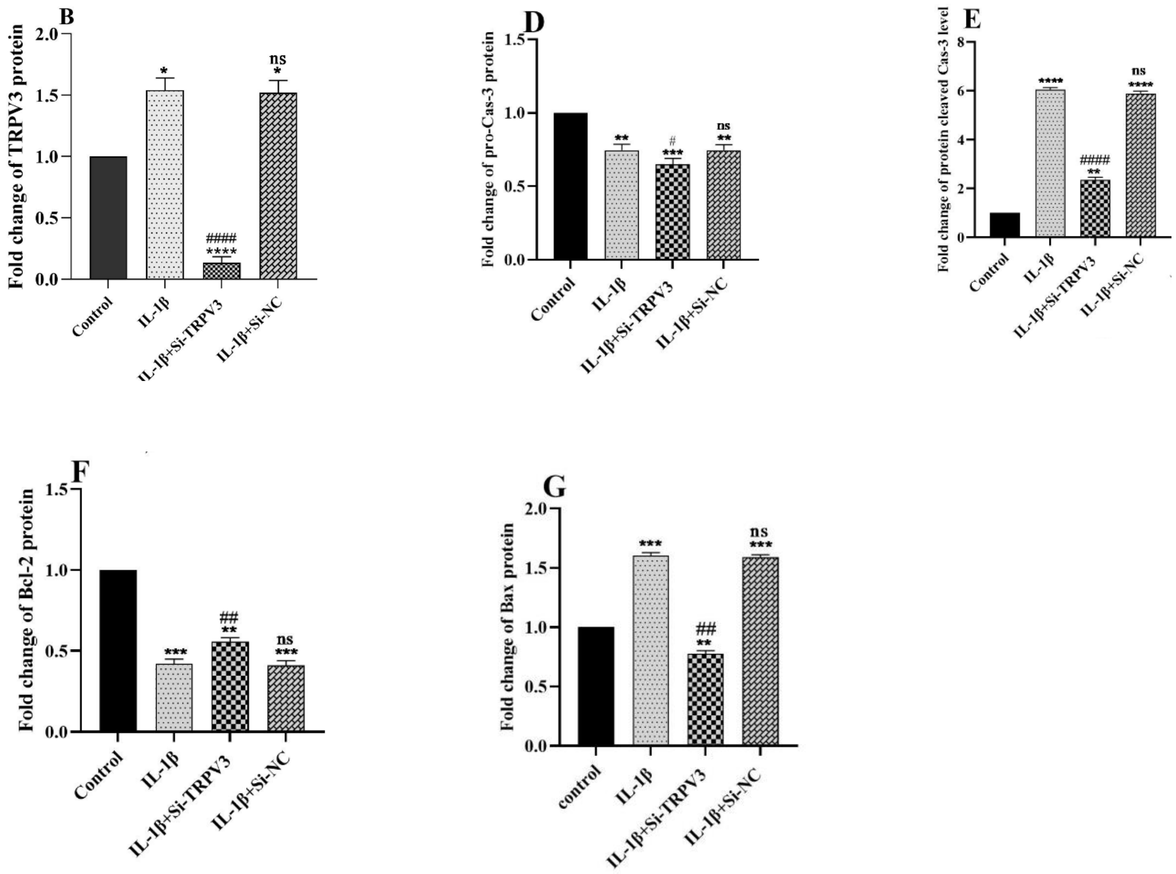

Figures 2, 5, and 6 (B, D, E, F, and G): Expression of TRPV3 in IL-1β treated C28/I2 chondrocyte cells and the effect of Si-TRPV3 on the protein expression of apoptosis and inflammation genes

Raw data to Figures 2, 5, and 6 (B, D, E, F, and G): The raw data indicating fold changes of studied genes of individual western blotting include TRPV3, Caspase-3, Bax, Bcl-2, iNOS and COX-2

Variable	Group	Target density	relative density =target density/loading control	relative density (fold of control)
			TRPV3/β-actin	TRPV3/β-actin (fold of control)
**TRPV3**	control	14224.146	0.806027715	1.00
	IL	28513.693	1.241099032	1.54
	IL+Si	2359.506	0.092680294	0.11
	IL+Si NC	27513.573	1.223828952	1.52
			Cas-3pro/β-actin	Cas-3pro/β-actin (fold of control)
**Cas-3pro**	control	21727.187	1.231196228	1.00
	IL	20545.693	0.894280502	0.73
	IL+Si	21012.551	0.825363195	0.67
	IL+Si NC	21084.522	0.937858869	0.76
			Cas-3cleaved/β-actin	Cas-3-cleaved/β-actin (fold of control)
**Cas-3cleaved**	control	2296.569	0.130137744	1.00
	IL	17965.028	0.781953388	6.01
	IL+Si	7807.489	0.306674524	2.36
	IL+Si NC	17269.467	0.768161725	5.90
			Bcl-2/β-actin	Bcl-2/β-actin (fold of control)
**Bcl-2**	control	18555.702	1.051480355	1.00
	IL	10191.066	0.443580638	0.42
	IL+Si	15113.874	0.593665916	0.56
	IL+Si NC	9676.635	0.430425597	0.41
			Bax/β-actin	Bax/β-actin (fold of control)
**Bax**	control	12407.823	0.703103668	1.00
	IL	25833.057	1.124420538	1.60
	IL+Si	13917.279	0.546664223	0.78
	IL+Si NC	25162.064	1.119231676	1.59
			iNOS/β-actin	iNOS/β-actin (fold of control)

EXCLI Journal 2025;24:325-338 – ISSN 1611-2156
Received: December 14, 2024, accepted: February 25, 2025, published: March 03, 2025

Variable	Group	Target density	relative density =target density/loading control	relative density (fold of control)
**iNOS**	control	13178.267	0.74676177	1.00
	IL	20071.752	0.873651546	1.17
	IL+Si	12252.075	0.481255787	0.64
	IL+Si NC	19479.075	0.866447115	1.16
			COX2/β-actin	COX-2/β-actin (fold of control)
**COX-2**	control	18265.288	1.035023709	1.00
	IL	28940.087	1.259658437	1.22
	IL+Si	18611.723	0.731059792	0.71
	IL+Si NC	27815.66	1.237266059	1.20
			.../β-actin	.../β-actin (fold of contrOL)
**β-actin**	control	17647.217		
	IL	22974.551		
	IL+Si	25458.551		
	IL+Si NC	22481.551		

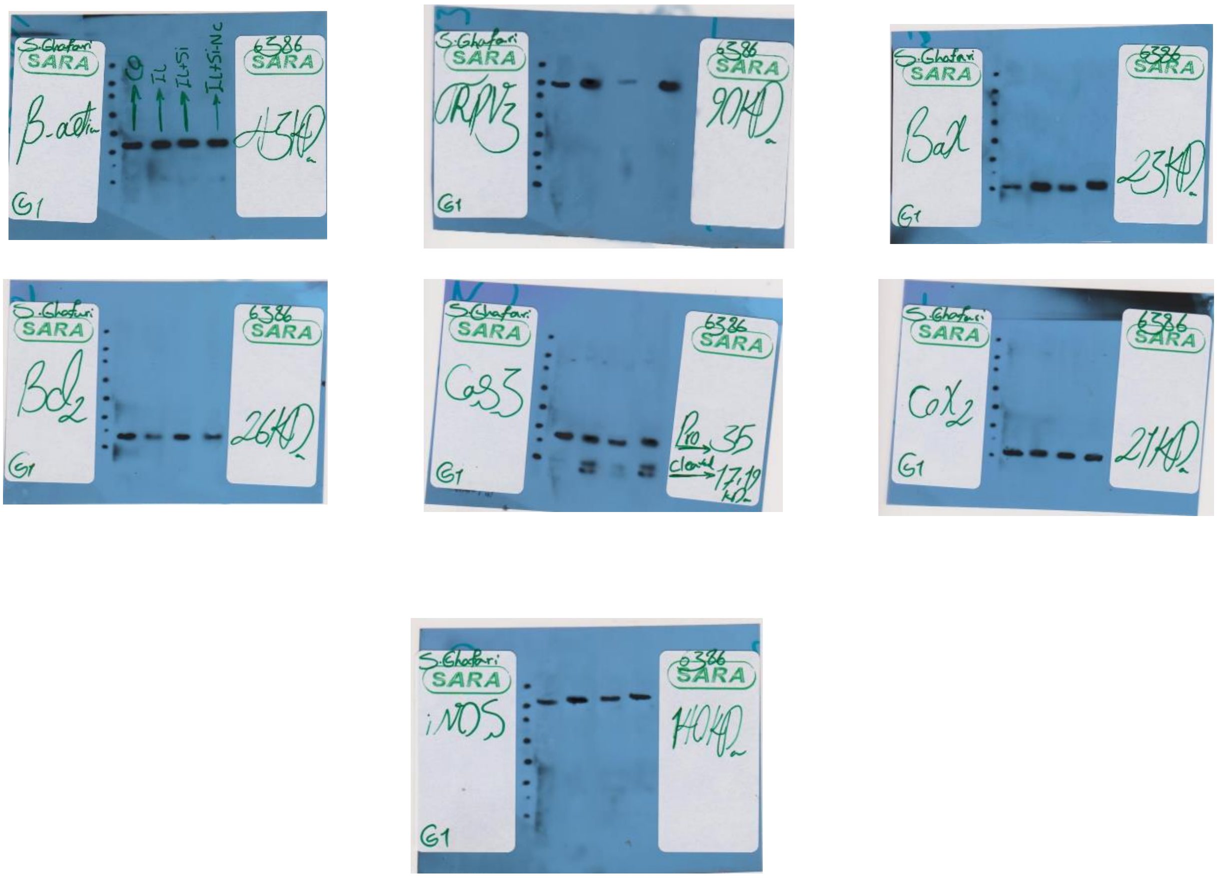

Raw data to Figures 2, 5, and 6 (C, H, and E): The captured image from the western blotting trial has been indicated.

EXCLI Journal 2025;24:325-338 – ISSN 1611-2156
Received: December 14, 2024, accepted: February 25, 2025, published: March 03, 2025

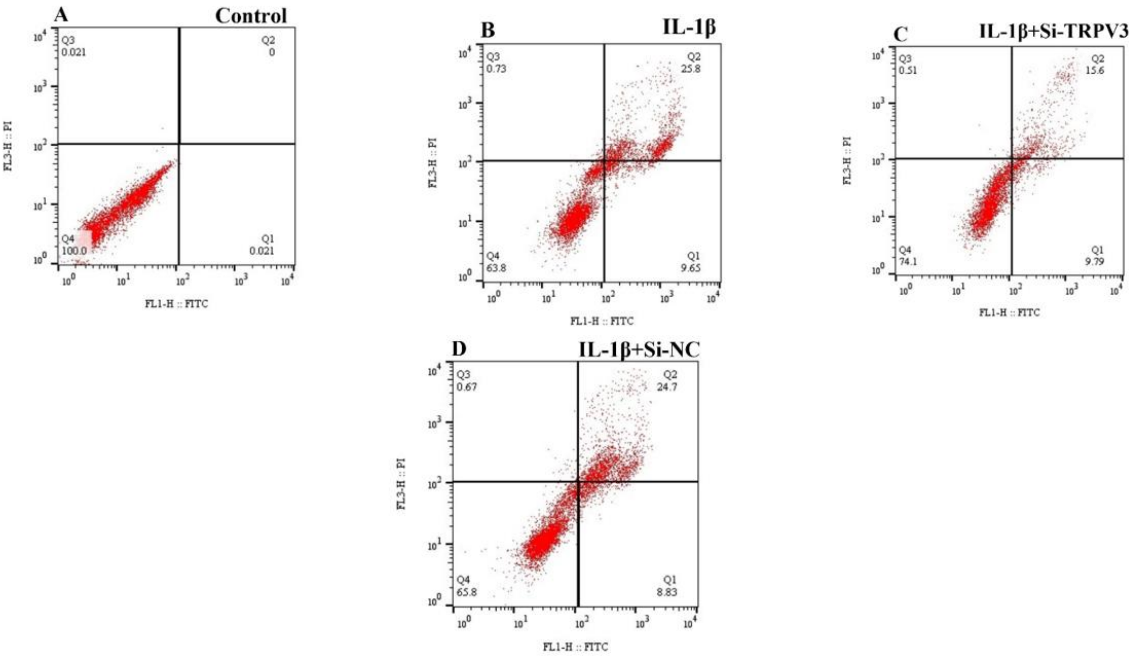

Figure 3 A, B, C, and D: Examination of cellular apoptosis via flow cytometry assay

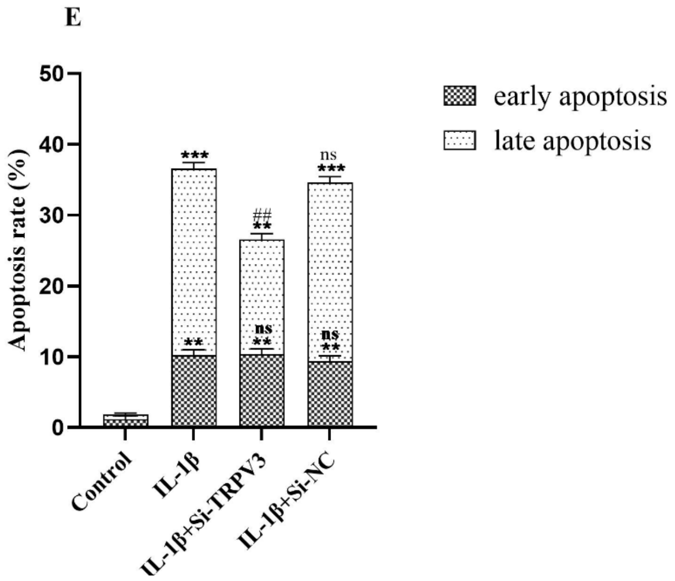

Figure 3E: The flow cytometry assessment of apoptosis using FITC-Annexin V and PI staining

Raw data to Figure 3E: Raw data of apoptosis based on Annexin V / PI

Cells	Early apoptotic cells (%)	Late apoptotic cells (%)	Early and late apoptosis, totally (%)
IL-1β	9.65	25.8	**35.45**
IL-1β+ Si-TRPV3	9.79	15.6	**25.39**
IL-1β+Si-NC	8.83	24.7	**33.53**
IL-1β vs control p-value	0.0050	0.0005	-
IL-1β+Si-TRPV3 vs control p-value	0.0048	0.0016	-
IL-1β+Si-NC vs control p-value	0.0059	0.00060	-
IL-1β+Si-TRPV3 vs IL-1β p-value	>0.05	0.0067	-
IL-1β+Si-NC vs IL-1β p-value	>0.05	>0.05	-

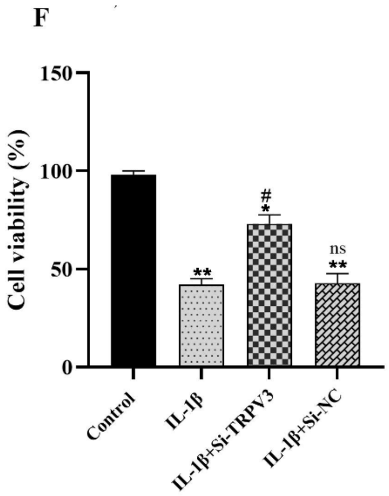

Figure 3F: Evaluated of cell viability via MTT test

Raw data to Figure 3F: the OD value of each well that measured via microplate reader at 490 nm

	**Control**	**IL-1β**	**IL-1β+ Si-TRPV3**	**IL-1β+Si-NC**	**blank**
**24h**	1.51	0.672	1.063	0.769	0.080
	1.472	0.639	1.188	0.682	0.080
	1.356	0.694	1.076	0.629	0.090

EXCLI Journal 2025;24:325-338 – ISSN 1611-2156
Received: December 14, 2024, accepted: February 25, 2025, published: March 03, 2025

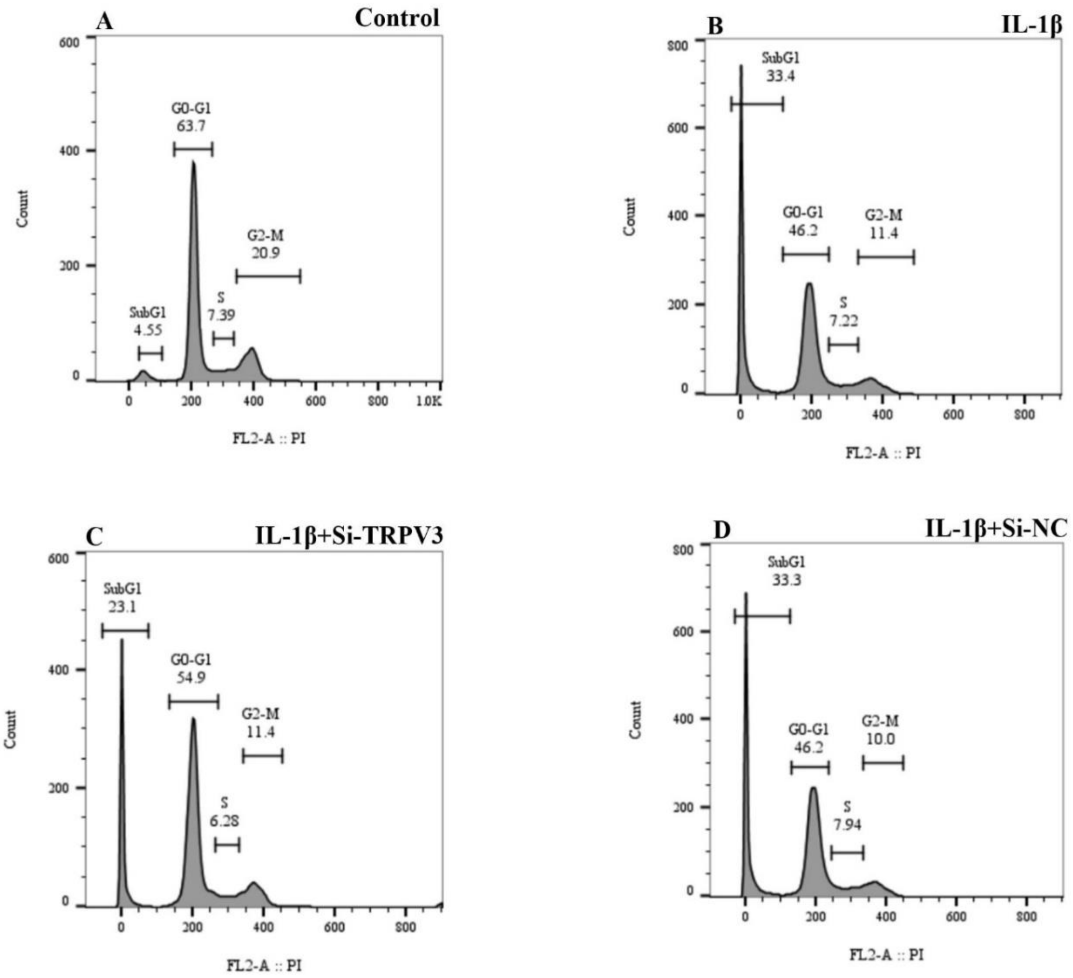

Figures 4 (A, B, C, and D): The flow cytometry diagrams for cells in different groups have been shown in this section. The data have been used to statistical analysis, and the results are shown in the following table.

Raw data to Figure 4 A, B, C, and D: The raw data shows the average of cell numbers in different cell cycle phases and also the result of statistical analysis.

Cell cycle phases	subG1	G0-G1	S	G2-M
Control	4.55%	63.7%	7.39%	**20.9%**
IL-1β	33.4%	46.2%	7.22%	**11.4%**
IL-1β+Si-TRPV3	23.1%	54.9%	6.28%	**11.4%**
IL-1β+Si-NC	33.3%	46.2%	7.94%	**10.0%**
IL-1β vs control p-value	<0.01	<0.05	-	**-**
IL-1β+Si-TRPV3 vs control p-value	<0.05	<0.05	-	**-**
IL-1β+Si-NC vs control p-value	<0.01	<0.05	-	**-**
IL-1β+Si-TRPV3 vs IL-1β p-value	<0.05	<0.05	-	**-**
IL-1β+Si-NC vs IL-1β p-value	>0.05	>0.05	-	**-**
